# Efficacy of reduced exertion high intensity interval training to modify cardiovascular disease-related risk factors—a narrative review

**DOI:** 10.3389/fresc.2026.1883275

**Published:** 2026-08-03

**Authors:** Todd A. Astorino, Riley Givens, Hayden Kunz, Matthew C. DeSouza

**Affiliations:** Department of Kinesiology, California State University—San Marcos, San Marcos, CA, United States

**Keywords:** blood pressure, body composition, cycling, high intensity interval training, mortality, sprint interval training, VO_2_max

## Abstract

Despite advances in medical imaging and treatment, cardiovascular disease (CVD) remains the primary cause of premature mortality in adults. Lifestyle modification including regular physical activity (PA) is a well recognized strategy to prevent onset of CVD, although the number of adults who attain recommended guidelines for PA is low, partially due to a perceived lack of time. Recent data show the efficacy of sprint interval training (SIT), brief, repeated “all-out” efforts performed at supramaximal intensities, to increase cardiorespiratory fitness (VO_2_max) in diverse groups of adults. This adaptation is important due to the known association between VO_2_max and all-cause mortality as well as mortality from CVD. A unique iteration of SIT is reduced exertion high intensity interval training (REHIT), which typically requires no more than 1 min of vigorous exercise consisting of two to three 20 s sprints within a 10 min exercise session. This review will define what REHIT is, explore its various health- and fitness-related benefits, and demonstrate its potential feasibility to modify cardiovascular disease-related risk factors in adults.

## 1 Introduction

Cardiovascular disease (CVD) represents various disorders affecting the heart and blood vessels, including coronary heart disease, heart failure, and stroke (1). As of 2023, CVD was identified as the primary cause of premature mortality in U. S. adults (915,973 deaths) and is significantly related to risk of obesity, hypertension, dyslipidemia, hyperglycemia, and low physical activity and unhealthy diet (2). The total health care costs of CVD are substantial and equal to $320 billion annually (3). Moreover, there is a dramatic loss of productivity attributed to CVD, as the annual economic cost is equal to $184 billion (4). Overall, CVD has profound effects on the physical and economic health of the United States and countries throughout the world.

One strategy repeatedly shown to have beneficial effects on CVD prevention is participation in regular physical activity (PA). Current PA guidelines developed by groups including the American College of Sports Medicine and American Heart Association recommend 150 min/wk of moderate intensity continuous training (MICT) combined with a minimum of 2 days per week of whole body resistance training to improve health status and reduce CVD risk. Much of this reduced risk attributed to regular PA lies in the significant improvements in cardiorespiratory fitness (VO_2_max) and glycemic control and reductions in blood lipids and blood pressure (BP) observed in response to various long-term PA interventions. Results from the Heritage Family Study (5) acquired in a large sample of healthy sedentary adults showed significant improvements in VO_2_max in response to 20 wk of supervised MICT, which were associated with concomitant increases in glucose homeostasis (6), high density lipoprotein (HDL) concentration (7) and reductions in blood pressure (BP) (8), percent body fat (%BF), waist to hip ratio (WHR), and visceral fat (9).

Despite the well-documented health benefits of MICT, only 25% of adults meet this guideline due to a perceived lack of time (10), which necessitates development of more time efficient PA regimens for use in adults. One promising alternative to MICT is high intensity interval training (HIIT), which consists of brief alternating high intensity efforts (30 s–4 min) separated by lower-intensity recovery (11). Although infinite iterations of HIIT exist, two widely-used protocols requiring submaximal efforts are the 10 × 1 min protocol (12) and the 4 × 4 min protocol (13). Prior data show their safety and efficacy to improve various health-based outcomes in healthy adults and those with existing disease; however, these protocols are not time-efficient as they require approximately 30 min including warm-up and recovery between efforts, which reduces their feasibility in most adults. Sprint interval training (SIT) characterized by supramaximal efforts including 4–6 “all-out” Wingate tests (14, 15) requires a low training volume of 2–3 min and significantly increases VO_2_max, cycling performance, and oxidative capacity, yet the need for extended recovery between sprints causes this regimen to require about 30 min to perform which is similar to HIIT. Moreover, the high volume and long sprint duration elicit marked feelings of fatigue and nausea which make this modality unpleasant and not feasible for the majority of adults.

A seminal study by Metcalfe et al. (16) required 15 inactive young men and women to undergo 18 sessions of reduced exertion high intensity interval training (REHIT), requiring one to two 10–20 s “all-out” sprints per session. Results showed significant increases in VO_2_max and insulin sensitivity, suggesting that this minimal dose of intense exercise equal to <40 s per session is adequate to improve metabolic health and aerobic capacity. Subsequent studies supported the efficacy of REHIT in various populations including inactive, overweight adults (17), adults with type 2 diabetes (T2D) (18), and inactive adults with elevated risk of CVD (19).

Three unique elements distinguish REHIT from SIT despite both modalities requiring brief, supramaximal efforts. First, a session of REHIT is ≤10 min and requires no more than 1 min of vigorous exercise, a lower training volume and session duration vs. SIT. Second, sprints are shorter (10–20 s) and typically performed at a lower resistance which elicits a lower peak power output than SIT. In turn, REHIT elicits lower rating of perceived exertion [RPE ∼ 13 = hard on the Borg (1982) 6–20 scale] vs. SIT (16, 20), which makes it seem less difficult and overall, may broaden its appeal to a wider range of adults.

Prior reviews concerning efficacy of REHIT on indices related to CVD risk focused on its effects on VO_2_max (21, 22) or combined REHIT with various SIT protocols (23), which are characterized by higher volume and overall, a longer session duration. A review is needed to comprehensively describe the potential of REHIT to impact cardiovascular disease-related risk factors through its effects on cardiorespiratory fitness, glycemic control, blood lipids, body composition, and BP.

The aim of this narrative review is to discuss how extremely low volume high intensity interval training, such as REHIT, can be used in CVD risk-factor modification in adults. Content presented will apply to clinicians and scientists who seek time efficient and effective forms of PA to improve health and fitness-related outcomes in their clients and potentially reduce future risk of CVD.

## 2 Depiction of studies included in this review and literature search methodology

Efficacy of REHIT has been repeatedly examined by scientists in Canada, the United Kingdom, and the United States. In these studies, up to three “all out” sprints are performed at durations ranging from 10 to 20 s per sprint. The total session duration of these REHIT protocols is up to 10 min, which requires less time than widely used HIIT and SIT protocols including the Norwegian 4 × 4 and repeated Wingate test regimens. To perform the literature search (completed from April 14 to May 1, 2026), we consulted various manuscripts already acquired by the Senior Author, examined references of these manuscripts, and completed a search on PubMed using the titles “REHIT,” “reduced exertion high intensity interval training,” 'sprint interval training,' and “intermittent exercise.”.

### 2.1 Effects of REHIT on VO_2_max

VO_2_max is not only recognized as a measure of cardiorespiratory fitness, but it is also related to endurance performance and has been identified as a clinical vital sign (24). For this reason, there are calls to incorporate testing of VO_2_max in primary care settings to predict future risk of all-cause mortality. Data from a meta-analysis by Kodama et al. (25) demonstrated that individuals with low VO_2_max exhibit substantially higher risk of all-cause mortality and CVD incidence vs. adults with average or high values, further supporting the strong association between VO_2_max and all-cause mortality. Numerous reviews have been published documenting the effect of HIIT and SIT on changes in VO_2_max (26, 27) as well as factors responsible for the change in VO_2_max induced by HIIT and SIT (28, 29); however, we recommend consulting these reviews for a more comprehensive evaluation of the overall effect of HIIT on improvements in VO_2_max.

Table 1 summarizes the change in VO_2_max reported in previous studies examining the effect of REHIT. In total, 338 participants have performed between 6 and 12 wk of REHIT, with the majority classified as insufficiently active. Only four studies employed a non-exercise control group (16, 17, 30, 31). Several studies compared the change in VO_2_max in response to REHIT to MICT (17–19), HIIT (32), or whole-body HIIT and SIT (31); however, the percent change reported in this table is the overall magnitude of increase in VO_2_max compared to the pre-training value. In all studies, there was a 2 min warm-up, 2–3 sprints of ≤20 s duration, and 3 min recovery between sprints and after the last sprint. Total training duration and session time were ≤ 1 min and ≤10 min. These results demonstrate that lower increases in VO_2_max occur in adults with T2D (18, 32) and in response to lower volumes of REHIT [6 wk, (30, 33, 34)]; whereas, more substantial increases in VO_2_max are revealed when healthy, inactive adults undergo 12 wk of REHIT [+ 9%–21%, respectively, (17, 35)]. Due to the known association between increases in VO_2_max and all-cause mortality (25), it is plausible that longer REHIT protocols ≥12 wk are preferred for further increasing VO_2_max and overall health status. Whether REHIT is superior for improving VO_2_max compared to MICT is unclear, as only a few studies have examined this topic. All of these studies comparing VO_2_max changes between REHIT and MICT are short-term and include a small number of participants, and only one had a control group. Aggregate data acquired from three studies in a total of 37 inactive adults suggest that REHIT elicits superior [(18, 19); 8 wk of REHIT] or similar increases in VO_2_max [ (17), 12 wk of REHIT] vs. MICT. This sizable increase in VO_2_max with REHIT is striking considering that during the 12 wk of training in the Gillen et al. (17) study, participants spent a total of 36 min (0.6 h) performing REHIT, compared to 27 h in those who underwent MICT. Overall, additional longer-term studies, including adequately-powered randomized controlled trials having larger sample sizes, are needed to better compare the VO_2_max response between REHIT and MICT.

**Table 1:** Studies demonstrating improvements in VO_2_max with REHIT.

Study	Duration (wk)	Sample and REHIT Frequency	REHIT	Participants	Control group	Change in VO_2_max
Metcalfe et al. (16)	6	8 women and 7 men	2 × 20 s	Sedentary	Yes	12%–15%
		3 sessions/wk				
Gillen et al. (33)	6	7 women and 7 men	3 × 20 s	Sedentary, overweight	No	12%
		3 sessions/wk				
Metcalfe et al. (63)	6	35 women and men	2 × 20 s	Sedentary	No	9%–10%
		3 sessions/wk				
Gillen et al. (17)	12	9 men	3 × 20 s	Sedentary	Yes	19%
		3 sessions/wk				
Revdal et al. (32)	12	9 men and women	2 × 20 s	Type 2 diabetes	No	5%
		3 sessions/wk				
Ruffino et al. (18)	8	16 men	2 × 20 s	Type 2 diabetes	No	7%
		3 sessions/wk				
Nalcakan et al. (36)	6	19 men and 17 women	2 × 10 or 2 × 20 s	Sedentary and active	No	10%
		3 sessions/wk				
Cuddy et al. (19)	8	Workplace-based intervention in 12 men and women	2 × 20 s	Inactive	No	12%
		2–4 sessions/wk				
Aspinall et al. (2020)	6	1 male 3 sessions/wk	2 × 20 s	Cystic fibrosis	No	6%
Metcalfe et al. (30)	6	Workplace-based intervention in 13 men and women	2 × 20 s	Inactive and active (*n* = 4)	Yes	7%
		2 sessions/wk				
Thomas et al. (34)	6	29 men and 13 women	2 × 20 s	Inactive	No	10%
		2 sessions/wk				
	6	3 sessions/wk	2 × 20 s			8%
	6	4 sessions/wk	2 × 20 s			7%
Bostad et al. (35)	12	6 men and 9 women	3 × 20 s	Untrained	No	21%
		3 sessions/wk				
Bostad et al. (42)	12	10 men and 10 women	3 × 20 s	Untrained	No	9%
		3 sessions/wk				
Hu et al. (31)	12	82 men 5 sessions/wk	2 × 20 s	Inactive	Yes	15%
Renwick et al. (71)	6	7 men and 10 women	3 × 20 s	Insufficiently active	No	17%
		3 sessions/wk				

REHIT is typically performed 3 d/wk, although data from Thomas et al. (34) demonstrated similar increases in VO_2_max at different frequencies of 2, 3, and 4 d/wk. Despite no apparent effect of training frequency on the change in VO_2_max, Nalcakan et al. (36) reported that REHIT consisting of two 10 s sprints for 6 wk led to a significantly lower increase in VO_2_max (4%) compared to a regimen requiring two 20 s sprints (10%), suggesting that a slightly longer sprint is preferred for optimizing the VO_2_max response. Although speculative, this is likely due to greater glycolytic flux, and in turn, rapid decreases in muscle glycogen and increases in glycolytic intermediates and blood lactate accumulation (BLa) characteristic of repeated, longer (30 s) Wingate-style sprints (37). This elevation in BLa is significant, as prior data (38) suggest that this is related to the adaptive response to exercise training.

**Figure 1:**
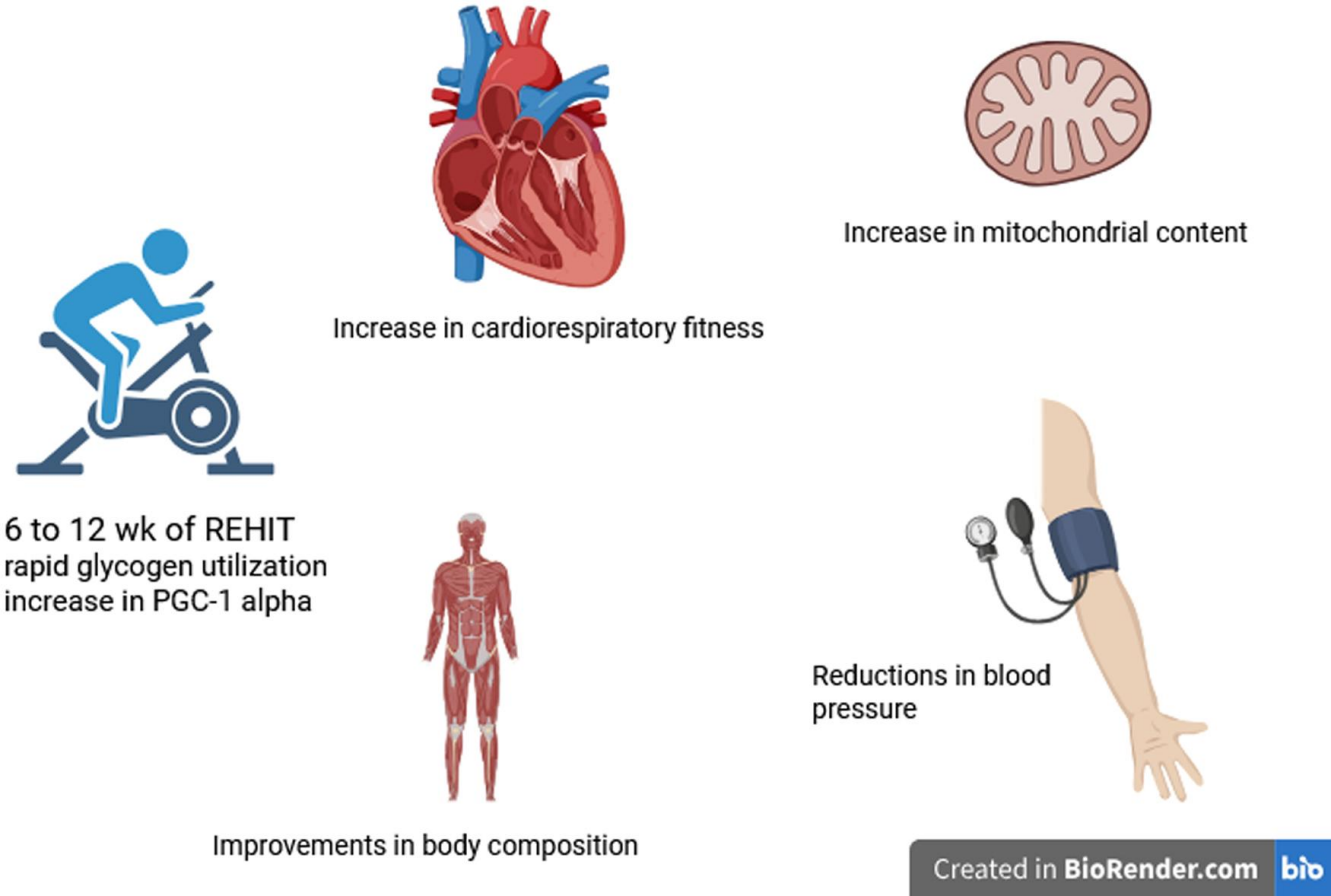
Proposed mechanisms and adaptations associated with REHIT.

The substantial perturbation associated with “all-out” sprints including REHIT also activates various transcription factors which are related to the adaptive response. Prior data show that a single 30 s sprint increases expression of 5′ AMP-activated protein kinase (AMPK) (39), an energy sensor in cells, and peroxisome proliferator-activated receptor-gamma coactivator 1-alpha (PGC1 alpha) (40) which is a marker of mitochondrial biogenesis. More recent data show that the 2 × 20 s REHIT protocol induced a 20% reduction in muscle glycogen and 16-fold increase in PGC1 alpha gene expression which likely activate adaptive signaling pathways (41). When performed long-term, these regular, robust metabolic perturbations enhance gene expression and along with increases in protein synthesis, likely potentiate the multitude of health- and fitness-related benefits associated with REHIT. In inactive adults, Gillen et al. (17) showed significant increases in citrate synthase activity and protein content of various subunits of the electron transport chain in response to 12 wk of 3 × 20 s REHIT, with these changes significantly different than a control group. Figure 1 shows various responses associated with chronic REHIT.

A widespread question posed in Exercise Physiology is what factors underpin the increase in VO_2_max observed with exercise training and whether they are central [e.g., oxygen (O_2_) delivery] or peripheral (O_2_ extraction) in nature. One study (35) examined the change in cardiac output (CO) in response to 12 wk of 3 × 20 s REHIT in 15 inactive men and women. VO_2_max increased by 21%, with significant increases seen at 2, 6, and 12 wk vs. baseline. Maximal CO was 6% higher in response to training, yet this increase only occurred at 12 wk, and there was no control group, casting doubt whether this increase in CO was in response to REHIT. In contrast, results showed a significant increase in arteriovenous O_2_ difference (avO_2_diff) at 2, 6, and 12 wk. In addition, an exploratory analysis showed that CO was significantly increased in men yet not women in response to REHIT. A follow-up study by this laboratory (42) demonstrated a lower relative increase in VO_2_max (9%) and no significant increase in CO in response to REHIT, meaning that the entire increase in VO_2_max was related to a significant increase in avO_2_diff. Prior studies implementing 4 or 6 wk of various “all-out” SIT regimens also revealed no change in CO [(43), 4–6 × 30 s; (44), 8 × 20 s at 170% VO_2_max], suggesting that the duration of training and the exact method of CO assessment may alter identification of factors mediating the observed increase in VO_2_max. Nevertheless, data from Mandic et al. (45) acquired in young healthy men and women (VO_2_max = 41 mL/kg/min) showed that 6 wk of 3 × 30 s SIT led to significant increases in VO_2_max (10%) which were attendant with increases in COmax, blood volume, plasma volume, and total Hb mass. Due to these conflicting results and only two small studies (35, 42) examining changes in CO and avO_2_diff, additional studies are needed to examine the mechanisms responsible for the widely-observed increase in VO_2_max in response to REHIT.

### 2.2 Effects of REHIT on body composition and blood pressure

Recent data (46) denote that 72.4% of adults in the United States are overweight according to body mass index (BMI), with the incidence of obesity (BMI > 30 kg/m^2^) being equal to 41.9%. Despite various results showing improved health status in obese adults with above-average VO_2_max (47), it is apparent that obesity is associated with elevated risk of hypertension, diabetes, stroke, and some forms of cancer (46) which contributes to substantial annual health care costs equal to $173 billion (48).

Body weight is altered by the balance between energy intake and expenditure, so PA must alter one of these elements to induce negative energy balance and in turn, sustained weight loss. Although total energy expenditure is difficult to quantify during REHIT due to a large contribution of nonoxidative metabolism, prior data show energy expenditure of 82 kcal during a 10 min session of REHIT and vigorous intensities as high as 95%HRmax (49), which reflect elevated energy expenditure compared to MICT. Moreover, there are reports of an elevation in VO_2_ after a session of REHIT, although this duration ranges from 15 to 45 min and only elicits an extra 33 kcal burned post-exercise (49). No study has examined changes in the appetite response after REHIT, yet Sim et al. (50) showed that SIT (repeated 15 s efforts at 170%VO_2_max) significantly reduces subsequent *ad libitum* food intake in inactive adults, which is associated with elevated blood glucose, BLa, and lower active ghrelin. Consequently, if REHIT elicits reductions in body mass or %BF, it may be due to attenuated food intake rather than a large energy expenditure.

A few studies (please see Table 2) demonstrate significant alterations in body mass and percent body fat (%BF) in response to REHIT, although not all data support its efficacy for improving body composition. In nine inactive men undergoing 12 wk of REHIT, Gillen et al. (17) showed a significant 2% decline in %BF which was different than the control group yet not different than changes observed in response to MICT. These data oppose those from an earlier study from this laboratory (33), showing no significant change in body mass in 14 overweight/obese adults in response to 18 sessions of REHIT. Potential explanations for this lack of effect may be the shorter exercise regimen, lack of %BF assessment, as well as the mixed sex sample of this study. In 12 inactive adults, no significant change in waist circumference (WC) was shown in response to 8 wk of 2 × 20 s REHIT (51), supporting the lack of changes in BMI or body mass observed in a seminal study (16). A small reduction in WC (−1.2 cm) was shown in response to 8 wk of REHIT performed by inactive adults with higher %BF (18), but this study lacked a control group. In 82 inactive men, significant, sizable reductions in body mass (−5 kg) and BMI were revealed compared to a control group in response to 12 wk of 5 d/wk of 2 × 20 s cycling separated by a brief 10 s passive recovery (31). Yet, dietary patterns were not considered in this study which questions the reliability of these findings. Overall, REHIT elicits modest changes in body mass and body composition, so higher volume exercise modes, including MICT and HIIT, are likely needed to induce more substantial changes in these outcomes in adults. In fact, Revdal et al. (32) showed a significant, albeit small reduction in %BF (−1.2%) in response to HIIT (10 × 1 min at 90%HRmax) which was not shown in response to REHIT (0.1%), despite the fact that participants undergoing REHIT had higher baseline BMI and %BF. Additional study is needed assessing potential changes in %BF and visceral fat in large samples of adults with class 2 obesity (BMI > 35 kg/m^2^) undergoing a minimum of 12 wk of REHIT, with statistical comparisons being made to those undergoing MICT, HIIT, or a non exercising control group.

**Table 2:** Studies demonstrating changes in blood pressure, blood lipids, glycemic control, and body composition in response to REHIT.

**Study**	**BP**	**Blood lipids**	**Glycemic control**	**Body mass**	**Body composition**
Metcalfe et al. (16)	NR	NR	No change in BG or insulin, ↑ IS in men	No change	No change in BMI
Gillen et al. (33)	↓ in SBP and MAP, no change in DBP	NR	No change in BG, ↓ insulin/HOMA-IR and ↑ in IS	No change	NR
Metcalfe et al. (63)	NR	NR	No change in BG, insulin, IS, or HOMA-IR outcomes	↑ in body mass	NR
Gillen et al. (17)	NR	NR	No change in BG, insulin, or HOMA-IR, ↑ in IS	No change	No change in BMI, ↓ in %BF
Revdal et al. (32)	↓ in SBP and DBP	NR	No change in BG or HOMA-IR	No change	No change in %BF, BMI, or WC
Ruffino et al. (18)	↓ in SBP, DBP, and MAP	No change in TG, LDL, or HDL	No change in BG, insulin, or OGTT measures	No change	No change in %BF
Nalcakan et al. (36)	NR	NR	NR	↑ in body mass	NR
Cuddy et al. (19)	↓ SBP, no change in DBP	↑ in HDL, ↓ in TG	No change in BG	No change	No change in %BF, ↓ in WC
Aspinall et al. (2020)	NR	NR	NR	No change	No change in BMI
Metcalfe et al. (30)	NR	NR	NR	No change	NR
Thomas et al. (34)	NR	NR	HR	No change	NR
Bostad et al. (35)	NR	NR	NR	No change	NR
Bostad et al. (42)	NR	NR	NR	No change	NR
Hu et al. (31)	↓ in SBP, DBP, and MAP	NR	NR	↓ in body mass	↓ in BMI
Renwick et al. (71)	NR	NR	NR	NR	NR

Changes (↓ or ↑) are compared to pre-training score and reflect significant differences (*p* < 0.05); NR, not reported; BP, blood pressure; BG, blood glucose; HOMA-IR, homeostatic model of insulin resistance; IS, insulin sensitivity; BMI, body mass index; SBP, systolic blood pressure; MAP, mean arterial pressure; DBP, diastolic blood pressure; WC, waist circumference; TG, triglycerides; LDL, low density lipoprotein; HDL, high density lipoprotein; OGTToral glucose tolerance test.

In 2025, new guidelines were published by the American Heart Association and American College of Cardiology denoting that healthy BP is less than 120/80 mm Hg, with hypertension being defined as values greater than 130–139/80–89 mm Hg. Observational data exhibit that 49.1% of adults in the U.S. have elevated BP (52), which is a known risk factor of CVD, type 2 diabetes, and stroke (53). Although regular PA is recommended to reduce BP in these patients, they are typically less active than adults without hypertension (54). A few studies demonstrate the effect of REHIT on BP in adults. In inactive, overweight/obese adults, significant reductions in resting systolic BP and mean arterial pressure (MAP) were revealed after 18 sessions of REHIT (33). Yet, data were not compared to a control group, and BP was determined 24 h after the last session of REHIT, so it is unclear if this is an acute or chronic effect of training. Significant declines in resting systolic BP were also shown by Cuddy et al. (19) in inactive adults with pre-hypertension (BP∼130/83 mm Hg), with this decline (−6 mm Hg) significantly greater than that seen in response to MICT (−2 mm Hg). Adults with healthy BP (115/84 mm Hg at baseline) also showed significant decreases in BP after undergoing 36 sessions of 2 × 20 s cycling (31). In obese men with type 2 diabetes, Ruffino et al. (18) showed significant reductions in systolic/diastolic BP and MAP in response to 8 wk of REHIT and MICT, although the study lacked a control group. Finally, Revdal et al. (32) showed reductions in DBP in nine overweight adults with type 2 diabetes and hypertension (SBP = 135/84 mm Hg) who underwent 12 wk of 2 × 20 s REHIT performed on a treadmill, yet this change was compared to the pre-training value rather than a control group.

The data reported above suggest a BP-lowering effect of REHIT in adults with healthy as well as elevated BP, suggesting a protective effect upon risk of CVD. However, these studies are characterized by small sample sizes, brief durations of training, and in many instances, no control group, so these results can be considered preliminary. To our knowledge, no study has identified mechanisms responsible for these changes observed in BP with REHIT. Nevertheless, changes in outcomes related to vascular function, which are associated with BP, were assessed in response to 6 wk of Wingate based SIT in inactive young adults (VO_2_max = 41 mL/kg/min) (55). Despite no significant change in BP, exercise training led to significant improvements in popliteal endothelial function and artery distensibility compared to pre-training, which may augment local nitric oxide availability and in turn, improve blood flow or reduce peripheral resistance. However, Bostad et al. (35) showed no effect of 12 wk of 3 × 20 s REHIT or endothelial function of arterial stiffness in healthy young adults. Overall, additional long-term studies are merited to examine efficacy of REHIT in large groups of adults with hypertension to identify the physiological factors responsible for this potential BP-lowering effect as well as determine any potential superiority of REHIT vs. MICT, HIIT, or a control condition.

### 2.3 Effects of REHIT on glycemic control and blood lipids

Physical activity is a primary regulator of onset of insulin resistance and T2D (56), which is why adults should perform 30 min/d of MICT and whole-body resistance training. Despite this, more than 35 million Americans have T2D and its prevalence has doubled since 2005 (1). Frequency of type 2 diabetes is greater in adults over 45 yr old due to aging-associated declines in muscle mass, increases in fat mass, as well as the responsibilities of work and family which may reduce participation in physical activity.

There are data (57) showing that lack of time is a potential barrier to PA in adults with T2D, making low-volume exercise such as REHIT a promising alternative to modify glucose control. Metcalfe et al. (58) showed no significant changes in glucose or insulin area under the curve (AUC) or insulin sensitivity in young adults (BMI = 23 kg/m^2^) 14 h after a single session of REHIT. Similarly, data from active men (age = 52 yr) showed no effect of a single bout of REHIT on 3 h post-prandial or 24 h glucose control (59). Nevertheless, Metcalfe et al. (60) reported significantly less time spent in hyperglycemia and attenuated 24 h blood glucose compared to no exercise in men with T2D, although these changes were small. In adults with stroke and T2D, Chen et al. (61) revealed that a single session of REHIT as well as MICT and HIIT significantly reduced mean blood glucose concentration post-exercise. Although preliminary, these data show the potential of REHIT to improve acute glycemic control in adults with hyperglycemia, although it has little effect in adults with euglycemia.

In inactive men performing six sessions of Wingate based SIT, Babraj et al. (62) showed significant reductions in insulin and glucose AUC as well as increased insulin sensitivity compared to pre-training. However, these findings were compared to pre-training data rather than to a non-exercise control group, making them preliminary. Nevertheless, this exercise regimen requiring as little as 16 min of vigorous exercise inspired subsequent studies showing efficacy of REHIT on glycemic control in adults. In nine sedentary men undergoing 36 sessions of 3 × 20 s REHIT, Gillen et al. (17) showed significant increases in the insulin sensitivity index and GLUT-4 protein content and significant reductions in glucose AUC vs. a control group. Metcalfe et al. (63) required sedentary adults (BMI = 25 kg/m^2^) to perform 18 sessions of REHIT. Results showed a trend for a reduction in insulin AUC, yet no significant change in the Cederholm index of insulin sensitivity. Ruffino et al. (18) examined changes in insulin sensitivity in men with T2D (BMI = 31 kg/m^2^) who underwent 8 wk of REHIT. Results showed no significant change in any measure vs. pre-training. In 12 men with T2D and stroke (age and HbA1C = 58 yr and 7.6%, respectively), 20 sessions of REHIT led to significant reductions in mean and peak blood glucose concentration and time in and above range acquired from continuous glucose monitoring vs. a control group (61). The disparate changes in measures related to glycemic control in these studies are likely associated with small sample sizes, different durations of REHIT, assessments used to measure changes in glycemic control, and dramatic individual responses to SIT. In fact, it is recommended that various sources of technical, biological, and random error be considered when examining potential training-induced changes in these outcomes in exercise training studies (64). Future studies should recruit larger samples of patients with pre-diabetes and T2D to account for this variability and better capture potential changes in glycemic control, including measures of HbA1C, in response to REHIT.

Recently, updated scientific guidelines were published for the management of dyslipidemia in adults (65). One recommendation is that lowering low density lipoprotein-C be a primary goal of therapy, as well as to treat dyslipidemia at an earlier age, especially in adults with family history of CVD or hyperlipidemia. Prior data from Fisher et al. (66) showed that 6 wk of MICT and HIIT significantly reduced blood lipid concentrations in inactive men with obesity, a finding supported by results shown in inactive women performing 16 wk of HIIT (67). However, less is known about the effect of SIT or REHIT on changes in blood lipids in adults. Freese et al. (68) demonstrated that 6 wk of SIT (4–8 × 30 s efforts) significantly reduced triglyceride concentrations in women at risk for metabolic syndrome. Cuddy et al. (19) showed significant reductions in triglyceride concentration and increased HDL in inactive adults who performed REHIT for 8 wk, whose magnitude was similar to that induced by MICT. Nevertheless, REHIT exhibited a significantly greater reduction in the metabolic syndrome z score than MICT, supporting its health-benefiting effects. Despite these results, a control group was not used in this study, so it is unclear if these differences are a direct result of exercise training. No significant effect of 8 wk of REHIT on blood lipids was also reported in adults with T2D (18). Additional long-term studies are needed to assess the potential of REHIT to improve the blood lipid profile and in turn, reduce risk of CVD in adults with dyslipidemia, as this group has not been included in studies investigating efficacy of REHIT.

### 2.4 Feasibility of REHIT

Concerns have been raised about the applicability and safety of “all-out” exercise including SIT and REHIT in inactive adults or those with existing disease (69). Yet, examination of all REHIT studies indicates that these protocols are safe for individuals without contraindications to MICT, as no adverse events have been reported other than dyspnea and nausea during the initial sessions. To reduce the metabolic stress and overall severity of fatigue in participants unaccustomed to REHIT, we recommend that the first few weeks of training require shorter sprint durations (10 and 15 s) as implemented in prior studies. In addition, recent data (20) showed that post-exercise enjoyment was significantly higher in response to REHIT compared to SIT as well as cycling at 110 percent ventilatory threshold. In addition, AUC for affective valence was significantly lower in response to REHIT, and 72% of participants (21/29) reported preference for this modality. These data are supported by no significant differences in affective valence or enjoyment between adults with average and below average VO_2_max who completed a single REHIT session (70), suggesting that adults with lower fitness do not perceive REHIT as aversive.

One existing issue related to the feasibility of REHIT is if adults can or will actually complete it in a free-living environment. For example, the majority of studies implementing REHIT were performed in a laboratory with supervision (16, 18, 33), which likely facilitates participation in these regimens. Nevertheless, two studies in inactive adults (19, 30) showed that REHIT can be performed in a workplace with high adherence, acceptability, and efficacy, as shown by high training compliance and significant increases in VO_2_max and various health-based outcomes. Additional long-term studies are needed to portray the acceptability and efficacy of REHIT in larger samples of adults completing REHIT at the workplace or at home.

### 2.5 Limitations of the evidence regarding efficacy of REHIT

Despite sizable interest in the efficacy of vigorous exercise to improve indices related to health and fitness in adults, the literature showing the potential of REHIT is relatively incomplete, as this review highlights findings from less than 20 peer-reviewed studies having relatively small sample sizes. Only four of these studies included a non-exercise control group, and the majority implemented REHIT for ≤ 8 wk and were performed in inactive adults rather than those with underlying disease. Moreover, the specific dose of REHIT used in these studies varied. In addition, no study has included participant follow-up after cessation of REHIT to understand if this modality is taken up in free-living adults, or if adaptations associated with participation in REHIT are rapidly lost upon training cessation. Therefore, our review demonstrates the potential of REHIT towards CVD risk-factor modification, yet additional studies are needed to truly show that it is effective to prevent CVD.

## 3 Conclusion

Reduced exertion high intensity interval training has been shown to be a more time efficient alternative to MICT and other modalities of HIIT. Results from this review demonstrate that 6–12 weeks of REHIT leads to sizable and significant increases in VO_2_max compared to baseline, with observed changes typically similar to MICT. REHIT elicits modest improvements in body composition and blood lipids, although it appears that more substantial effects on BP and glycemic control occur in adults with and without underlying disease. The overall feasibility of REHIT has been shown in several studies, as it does not lead to adverse events and is well tolerated when performed at home, in a workplace, or when supervised in a laboratory setting. However, only four studies included in this review compared adaptations observed in response to REHIT to a control group, which requires initiation of large-scale, longer-term randomized controlled trials. Moreover, many studies are characterized by small sample size and brief training programs, reducing the quality of these results. In addition, we acknowledge that the varied cardiometabolic benefits of REHIT are indirect, and no study has actually determined frequency of cardiovascular events or CVD incidence in response to long-term administration of REHIT. Consequently, additional studies are needed in very large samples of inactive adults and those at risk for and living with CVD to further validate the true efficacy and applicability of REHIT for preventing CVD.

## References

[1] National Center for Health Statistics. Multiple Cause of Death 2018-2023 on CDC WONDER Database. Available online at: https://wonder.cdc.gov (Accessed May 1, 2026).

[2] Palaniappan LP Allen NB Almarzooq ZI Anderson CAM Arora P Avery CL; American Heart Association Council on Epidemiology and Prevention Statistics Committee and Stroke Statistics Committee. 2026 Heart disease and stroke statistics: a report of US and global data from the American Heart Association. Circulation. (2026) 153(9):e275–e906. 10.1007/s00421-018-3980-2 41562125

[3] Tsao CW Aday AW Almarzooq ZI Anderson CAM Arora P Avery CL. Heart disease and stroke statistics—2023 Update: a report from the American Heart Association. Circulation. (2023) 147, e93–e621. 10.1161/CIR.0000000000001123 36695182 PMC12135016

[4] Gordois AL Toth PP Quek RG Proudfoot EM Paoli CJ Gandra SR. Productivity losses associated with cardiovascular disease: a systematic review. Expert Rev Pharmacoecon Outcomes Res. (2016) 16(6):759–69. 10.1080/14737167.2016.1259571 27831848

[5] Bouchard C An P Rice T Skinner JS Wilmore JH Gagnon J. Familial aggregation of VO(_2_max) response to exercise training: results from the HERITAGE family study. J Appl Physiol. (1999) 87(3):1003–8. 10.1152/jappl.1999.87.3.1003 10484570

[6] Boulé NG Weisnagel SJ Lakka TA Tremblay A Bergman RN Rankinen T. HERITAGE Family Study. Effects of exercise training on glucose homeostasis: the HERITAGE family study. Diabetes Care. (2005) 28(1):108–14. 10.2337/diacare.28.1.108 15616242

[7] Leon AS Rice T Mandel S Despres J-P Bergeron J Gagnon J. Blood lipid response to 20 weeks of supervised exercise in a large biracial population: the HERITAGE family study. Metab Clin Exp. (2000) 49(4):513–20. 10.1016/s0026-0495(00)80018-9 10778878

[8] Wilmore J Stanforth P Gagnon J Rice T Mandel S Leon A. Heart rate and blood pressure changes with endurance training: the HERITAGE family study. Med Sci Sports Exerc. (2001) 33(1):107–16. 10.1097/00005768-200101000-00017 11194095

[9] Wilmore JH Després J-P Stanforth PR Mandel S Rice T Gagnon J. Alterations in body weight and composition consequent to 20 wk of endurance training: the HERITAGE family study. Am J Clin Nutr. (1999) 70(3):346–52. 10.1093/ajcn/70.3.346 10479196

[10] Kiens B Beyer N Brage S Hyldstrup L Ottesen LS Overgaard K. Fysisk inaktivitet–konsekvenser og sammenhaenge [physical inactivity–consequences and correlations]. Ugeskr Laeger. (2007) 169(25):2442–5. 17594841

[11] Coates AM Joyner MJ Little JP Jones AM Gibala MJ. A perspective on high-intensity interval training for performance and health. Sports Med. (2023) 53(S1):85–96. 10.1007/s40279-023-01938-6 37804419 PMC10721680

[12] Little JP Safdar A Wilkin GP Tarnopolsky MA Gibala MJ. A practical model of low-volume high-intensity interval training induces mitochondrial biogenesis in human skeletal muscle: potential mechanisms. J Physiol. (2010) 588(6):1011–22. 10.1113/jphysiol.2009.181743 20100740 PMC2849965

[13] Wisløff U Støylen A Loennechen JP Bruvold M Rognmo Ø Haram PM. Superior cardiovascular effect of aerobic interval training versus moderate continuous training in heart failure patients: a randomized study. Circulation. (2007) 115(24):386–94. 10.1161/CIRCULATIONAHA.106.675041 17548726

[14] Burgomaster KA Heigenhauser GJ Gibala MJ. Effect of short-term sprint interval training on human skeletal muscle carbohydrate metabolism during exercise and time-trial performance. J Appl Physiol. (2006) 100(6):2041–7. 10.1152/japplphysiol.01220.2005 16469933

[15] Astorino TA Allen RP Roberson DW Jurancich M Lewis R McCarthy K. Adaptations to high-intensity interval training are independent of gender. Eur J Appl Physiol. (2011) 111(7):1279–86. 10.1007/s00421-010-1741-y 21132441

[16] Metcalfe RS Babraj JA Fawkner S Vollaard NBJ. Towards the minimal amount of exercise for improving metabolic health: beneficial effects of reduced-exertion high-intensity interval training. Eur J Appl Physiol. (2012) 112(7):2767–75. 10.1007/s00421-011-2254-z 22124524

[17] Gillen JB Martin BJ MacInnis MJ Skelly LE Tarnopolsky MA Gibala MJ. Twelve weeks of sprint interval training improves indices of cardiometabolic health similar to traditional endurance training despite a five-fold lower exercise volume and time commitment. PLoS One. (2016) 11(4):e0154075. 10.1371/journal.pone.0154075 27115137 PMC4846072

[18] Ruffino JS Songsorn P Haggett M Edmonds D Robinson AM Thompson D. A comparison of the health benefits of reduced-exertion high-intensity interval training (REHIT) and moderate-intensity walking in type 2 diabetes patients. Appl Physiol Nutr Metab. (2018) 42(2):202–8. 10.1139/apnm-2016-0497 28121184

[19] Cuddy JS Ramos JS Dalleck LC. Reduced exertion high-intensity interval training is more effective at improving cardiorespiratory fitness and cardiometabolic health than traditional moderate-intensity continuous training. Int J Environ Res Publ Health. (2019) 16(3):483. 10.3390/ijerph16030483 PMC6388288 30736402

[20] Kinghorn DJ Hutchinson M Curry S Zhang J Vollaard NBJ. When moderate becomes unpleasant and intense is manageable: exercise intensity and duration interact to regulate exercise enjoyment and changes in affective valence. J Sport Exerc Psychol. (2026) 48(1):35–45. 10.1123/jsep.2025-0086 41569868

[21] Vollaard NBJ Metcalfe RS. Research into the health benefits of sprint interval training should focus on protocols with fewer and shorter sprints. Sports Med. (2017) 47(12):2443–51. 10.1007/s40279-017-0727-x 28391489 PMC5684281

[22] Vollaard NBJ Metcalfe RS Williams S. Effect of number of sprints in an SIT session on change in VO_2_max: a meta-analysis. Med Sci Sports Exerc. (2017) 49(6):1147–56. 10.1249/MSS.0000000000001204 28079707

[23] Bi Z Yin M Xu K Marcotte-Chénard A Zhong Y Gu Z. One size does not fit all: a meta-analysis of 115 trials comparing high-intensity interval and moderate-to-vigorous-intensity continuous training across diverse participants, protocols, and outcomes. Scand J Med Sci Sports. (2026) 36(3):370243. 10.1111/sms.70243 41804294

[24] Hanscombe KB Persyn E Traylor M Glanville KP Hamer M Coleman JRI. The genetic case for cardiorespiratory fitness as a clinical vital sign and the routine prescription of physical activity in healthcare. Genome Med. (2021) 13(1):180. 10.1186/s13073-021-00994-9 34753499 PMC8579601

[25] Kodama S Saito K Tanaka S Maki M Yachi Y Asumi M. Cardiorespiratory fitness as a quantitative predictor of all-cause mortality and cardiovascular events in healthy men and women: a meta-analysis. J Amer Med Assoc. (2009) 301(19):2024–35. 10.1001/jama.2009.681 19454641

[26] Sloth M Sloth D Overgaard K Dalgas U. Effects of sprint interval training on VO_2_max and aerobic exercise performance: a systematic review and meta-analysis. Scand J Med Sci Sports. (2013) 23(6):e341–52. 10.1111/sms.12092 23889316

[27] Weston M Taylor KL Batterham AM Hopkins WG. Effects of low-volume high-intensity interval training (HIT) on fitness in adults: a meta-analysis of controlled and non-controlled trials. Sports Med. (2014) 44(7):1005–17. 10.1007/s40279-014-0180-z 24743927 PMC4072920

[28] Astorino TA Causer E Hazell TJ Arhen BB Gurd BJ. Change in central cardiovascular function in response to intense interval training: a systematic review and meta-analysis. Med Sci Sports Exerc. (2022) 54(12):1991–2004. 10.1249/MSS.0000000000002993 35881924

[29] Rosenblat MA Granata C Thomas SG. Effect of interval training on the factors influencing maximal oxygen consumption: a systematic review and meta-analysis. Sports Med. (2022) 52(6):1329–52. 10.1007/s40279-021-01624-5 35041180

[30] Metcalfe RS Atef H Mackintosh K McNarry M Ryde G Hill DM. Time-efficient and computer-guided sprint interval exercise training for improving health in the workplace: a randomised mixed-methods feasibility study in office-based employees. BMC Public Hlth. (2020) 20(1):313. 10.1186/s12889-020-8444-z PMC7068982 32164631

[31] Hu M Chen X Nie J Shi Q Kong Z. Real-world efficacy of equipment-free reduced-exertion high-intensity interval training in improving physical and mental health in inactive males: a randomized controlled trial. J Exerc Sci Fit. (2025) 23(4):273–83. 10.1016/j.jesf.2025.06.006 40689309 PMC12273568

[32] Revdal A Hollekim-Strand SM Ingul CB. Can time efficient exercise improve cardiometabolic risk factors in type 2 diabetes? A pilot study. J Sports Sci Med. (2016) 15(2):308–13. 27274669 PMC4879445

[33] Gillen JB Percival ME Skelly LE Martin BJ Tan RB Tarnopolsky MA. Three minutes of all-out intermittent exercise per week increases skeletal muscle oxidative capacity and improves cardiometabolic health. PLoS One. (2014) 9(11):e111489. 10.1371/journal.pone.0111489 25365337 PMC4218754

[34] Thomas G Songsorn P Gorman A Brackenridge B Cullen T Fitzpatrick B. Reducing training frequency from 3 or 4 sessions/week to 2 sessions/week does not attenuate improvements in maximal aerobic capacity with reduced-exertion high-intensity interval training (REHIT). Appl Physiol Nutr Metab. (2020) 45(6):683–5. 10.1139/apnm-2019-0750 32078337

[35] Bostad W Valentino SE McCarthy DG Richards DL MacInnis MJ MacDonald MJ. Twelve weeks of of sprint interval training increases peak cardiac output in previously untrained individuals. Eur J Appl Physiol. (2021) 21(9):2449–58. 10.1007/s00421-021-04714-4 34014402

[36] Nalçakan GR Songsorn P Fitzpatrick BL Yüzbasioglu Y Brick NE Metcalfe RS. Decreasing sprint duration from 20 to 10 s during reduced-exertion high-intensity interval training (REHIT) attenuates the increase in maximal aerobic capacity but has no effect on affective and perceptual responses. Appl Physiol Nutr Metab. (2017) 43(4):338–44. 10.1139/apnm-2017-0597 29172029

[37] Parolin ML Chesley A Matsos MP Spriet LL Jones NL Heigenhauser GJF. Regulation of skeletal muscle glycogen phosphorylase and PDH during maximal intermittent exercise. Am J Physiol Endocrinol Metab. (1999) 277:E890–900. 10.1152/ajpendo.1999.277.5.E890 10567017

[38] Egan B Zierath JR. Exercise metabolism and the molecular regulation of skeletal muscle adaptation. Cell Metab. (2013) 17:162–84. 10.1016/j.cmet.2012.12.012 23395166

[39] Fuentes T Guerra B Ponce-González JG Morales-Alamo D Guadalupe-Grau A Olmedillas H. Skeletal muscle signaling response to sprint exercise in men and women. Eur J Appl Physiol. (2012) 112:1917–27. 10.1007/s00421-011-2164-0 21928060

[40] Guerra B Guadalupe-Grau A Fuentes T Ponce-González JG Morales-Alamo D Olmedillas H. SIRT1, AMP-activated protein kinase phosphorylation and downstream kinases in response to a single bout of sprint exercise: influence of glucose ingestion. Eur J Appl Physiol. (2010) 109:731–43. 10.1007/s00421-010-1413-y 20217115

[41] Metcalfe RS Koumanov F Ruffino JS Stokes KA Holman GD Thompson D. Physiological and molecular responses to an acute bout of reduced-exertion high-intensity interval training (REHIT). Eur J Appl Physiol. (2015) 115(11):2321–34. 10.1007/s00421-015-3217-6 26156806

[42] Bostad W Williams JS Van Berkel EK Richards DL MacDonald MJ Gibala MJ. Biological sex does not influence the peak cardiac output response to twelve weeks of sprint interval training. Sci Rep. (2023) 13:22996. 10.1038/s41598-023-50016-4 38151488 PMC10752867

[43] Macpherson RE Hazell TJ Olver TD Paterson DH Lemon PW. Run sprint interval training improves aerobic performance but not maximal cardiac output. Med Sci Sports Exerc. (2011) 43(1):115–22. 10.1249/mss.0b013e3181e5eacd 20473222

[44] Raleigh JP Giles MD Islam H Nelms M Bentley RF Jones JH. Contribution of central and peripheral adaptations to changes in maximal oxygen uptake following 4 weeks of sprint interval training. Appl Physiol Nutr Metab. (2018) 43(10):1059–68. 10.1139/apnm-2017-0864 29733694

[45] Mandić M Hansson B Lovrić A Sundblad P Vollaard N Lundberg T. Improvements in maximal oxygen uptake after sprint-interval training coincide with increases in central hemodynamic factors. Med Sci Sports Exerc. (2022) 54(6):944–52. 10.1249/MSS.0000000000002872 35136000

[46] Bryan S Afful J Carroll M Te-Ching C Orlando D Fink S. National health and nutrition examination survey 2017–march 2020 prepandemic data files development of files and prevalence estimates for selected health outcomes. Natl Health Stat Report. (2021) 158:1–21. 10.15620/cdc:106273 PMC11513744 39380201

[47] Barry VW Baruth M Beets MW Durstine JL Liu J Blair SN. Fitness vs. Fatness on all-cause mortality: a meta-analysis. Prog Cardiovasc Dis. (2014) 56(4):382–90. 10.1016/j.pcad.2013.09.002 24438729

[48] Ward ZJ Bleich S Long MW Gortmaker SL. Association of body mass index with health care expenditures in the United States by age and sex. PLoS One. (2021) 16(3):e0247307. 10.1371/journal.pone.0247307 33760880 PMC7990296

[49] Clausen RD Astorino TA. Excess post-exercise oxygen consumption after reduced exertion high-intensity interval training on the cycle ergometer and rowing ergometer. Eur J Appl Physiol. (2024) 124(3):815–25. 10.1007/s00421-023-05309-x 37787925

[50] Sim AY Wallman KE Fairchild TJ Guelfi KJ. High-intensity intermittent exercise attenuates ad-libitum energy intake. Int J Obes. (2014) 38(3):217–22. 10.1038/ijo.2013.102 23835594

[51] Leahy DJ Dalleck LC Ramos JS. Changes in the fitness fatness Index following reduced exertion high-intensity interval training versus moderate-intensity continuous training in physically inactive adults. Front Sports Act Living. (2022) 4:961957. 10.3389/fspor.2022.961957 35992158 PMC9388827

[52] National Center for Health Statistics. High Blood Pressure. U.S. Department of Health and Human Services, Centers for Disease Control and Prevention (2026).

[53] Whelton PK Carey RM Aronow WS Casey DE Collins KJ Dennison Himmelfarb C. 2017 ACC/AHA/AAPA/ABC/ACPM/AGS/APhA/ASH/ASPC/NMA/PCNA guideline for the prevention, detection, evaluation, and management of high blood pressure in adults: a report of the American College of Cardiology/American Heart Association task force on clinical practice guidelines. Hypertension. (2018) 71(6):e13–e115. 10.1161/HYP.0000000000000065 29133356

[54] Churilla JR Ford ES. Comparing physical activity patterns of hypertensive and nonhypertensive US adults. Am J Hypertens. (2010) 23(9):987–93. 10.1038/ajh.2010.88 20431526

[55] Rakobowchuk M Tanguay S Burgomaster KA Howarth KR Gibala MJ MacDonald MJ. Sprint interval and traditional endurance training induce similar improvements in peripheral arterial stiffness and flow-mediated dilation in healthy humans. Am J Physiol. (2008) 295(1):R236–42. 10.1152/ajpregu.00069.2008 PMC2494806 18434437

[56] Fiuza-Luces C Santos-Lozano A Joyner M Carrera-Bastos P Picazo O Zugaza JL. Exercise benefits in cardiovascular disease: beyond attenuation of traditional risk factors. Nat Rev Cardiol. (2018) 15(12):731–43. 10.1038/s41569-018-0065-1 30115967

[57] Hoare E Stavreski B Jennings GL Kingwell BA. Exploring motivation and barriers to physical activity among active and inactive Australian adults. Sports. (2017) 5(3):47. 10.3390/SPORTS5030047 29910407 PMC5968958

[58] Metcalfe RS Fawkner S Vollaard NBJ. No acute effect of reduced-exertion high-intensity interval training (REHIT) on insulin sensitivity. Int J Sports Med. (2016) 37(5):354–8. 26855434 10.1055/s-0035-1564258

[59] Chaudhry BA Morrell JS Cook SB Goodwin NJ Brian MS. Reduced-exertion high-intensity interval training (REHIT) provides limited improvement in post-prandial and 24-hour glycemia in healthy, physically active men. J Ex Sci Fit. (2026) 24:200452. 10.1016/j.jesf.2026.200452 PMC12930057 41743231

[60] Metcalfe RS Fitzpatrick B Fitzpatrick S McDermott G Brick N McClean C. Extremely short duration interval exercise improves 24-h glycaemia in men with type 2 diabetes. Eur J Appl Physiol. (2018) 118(12):2551–62. 10.1007/s00421-018-3980-2 30171349 PMC6244655

[61] Chen K Ma H Wang Y Li D Li J Huang Y. Effects of three aerobic exercise protocols on acute glucose response and continuous glucose monitoring trends in patients with type 2 diabetes mellitus and stroke: a randomized controlled trial. BMC Sports Sci Med Rehabil. (2025) 17:359. 10.1186/s13102-025-01402-0 41316479 PMC12661827

[62] Babraj JA Vollaard NB Keast C Guppy FM Cottrell G Timmons JA. Extremely short duration high intensity interval training substantially improves insulin action in young healthy males. BMC Endocrin Disord. (2009) 9:3. 10.1186/1472-6823-9-3 PMC2640399 19175906

[63] Metcalfe RS Tardif N Thompson D Vollaard NBJ. Changes in aerobic capacity and glycaemic control in response to reduced-exertion high-intensity interval training (REHIT) are not different between sedentary men and women. Appl Physiol Nutr Metab. (2016) 41(11):1117–23. 10.1139/apnm-2016-0253 27753506

[64] Metcalfe RS Gurd BJ Vollaard NBJ. Exploring interindividual differences in fasting and postprandial insulin sensitivity adaptations in response to sprint interval exercise training. Eur J Sports Sci. (2023) 23(9):1950–60. 10.1080/17461391.2022.2124385 36093904

[65] Blumenthal RS Morris PB Gaudino M Johnson HM Anderson TS Bittner VA. 2026 ACC/AHA/AACVPR/ABC/ACPM/ADA/AGS/APhA/ASPC/NLA/PCNA guideline on the management of dyslipidemia: a report of the American College of Cardiology/American Heart Association joint committee on clinical practice guidelines. J Am Coll Cardiol. (2026) 87:2624–757. 10.1016/j.jacc.2025.11.016 41824590

[66] Fisher G Brown AW Bohan Brown MM Alcorn A Noles C Winwood L. High intensity interval- vs moderate intensity- training for improving cardiometabolic health in overweight or obese males: a randomized controlled trial. PLoS One. (2015) 10(10):e0138853. 10.1371/journal.pone.0138853 26489022 PMC4619258

[67] Alvarez C Ramirez-Campillo R Martinez-Salazar C Castillo A Gallardo F Ciolac EG. High-intensity interval training as a tool for counteracting dyslipidemia in women. Int J Sports Med. (2018) 39(5):397–406. 10.1055/s-0044-100387 29564840

[68] Freese EC Gist NH Acitelli RM McConnell WJ Beck CD Hausman DB. Acute and chronic effects of sprint interval exercise on postprandial lipemia in women at-risk for the metabolic syndrome. J Appl Physiol. (2015) 118(7):872–9. 10.1152/japplphysiol.00380.2014 25593284

[69] Hardcastle SJ Ray H Beale L Hagger MS. Why sprint interval training is inappropriate for a largely sedentary population. Front Psychol. (2014) 5(5):1505. 10.3389/fpsyg.2014.01505 25566166 PMC4274872

[70] Astorino TA Clausen RDC Marroquin J Arthur B Stiles K. Similar perceptual responses to reduced exertion high intensity interval training (REHIT) in adults differing in cardiorespiratory fitness. Physiol Behav. (2020) 213:112687. 10.1016/j.physbeh.2019.112687 31622613

[71] Renwick JRM Crukley J Kudsi M Binet ER Bone J Mulkewich NJ. Six weeks of low-volume sprint interval training improves peak oxygen uptake compared to a non-exercise control: a randomized controlled trial. Scand J Med Sci Sports. (2025) 35(9):e70130. 10.1111/sms.70130 40879181 PMC12395895

